# Public Knowledge, Attitude, and Practice on Herbal Remedies Used During Pregnancy and Lactation in West Bank Palestine

**DOI:** 10.3389/fphar.2020.00046

**Published:** 2020-02-14

**Authors:** Ahmad M. Eid, Nidal Jaradat

**Affiliations:** Department of Pharmacy, Faculty of Medicine and Health Sciences, An-Najah National University, Nablus, Palestine

**Keywords:** medicinal plants, pregnancy, lactation, public health, ethnopharmacology

## Abstract

The use of herbal products by pregnant and lactating mothers without awareness of their harmful effects may expose both mother and fetus or infant to great dangers, such as abortion, premature delivery, uterine bleeding, and physical and mental retardation of the fetus. Thus, the aim of this study was to investigate the extent to which herbal product treatment is used and the reason for which such products are used and to ensure that these reasons are correct. An ethnopharmacological survey (cross-sectional observational design study) using a pre-piloted questionnaire was undertaken on herbal products used by pregnant and lactating women in the West Bank area of Palestine. A questionnaire was distributed to 350 pregnant and lactating women. The informed consent forms, ethics, and aims of the present study were reviewed and approved by the Institutional Review Board (IRB) at An-Najah National University. To identify the most important species used, the use value (UV) index was employed, while the SPSS program was used to analyze the data. Collected data revealed that 13 medicinal plants are utilized, while 12 plants are not used during pregnancy. Moreover, 15 plants are utilized and 9 plants are not used during lactation for treating and dealing with various problems. The most commonly used plants belonged to 14 families, including Lamiaceae, Apiaceae, Leguminosae, and Rubiaceae. The plants most used during pregnancy were sage (*Salvia fruticosa*), anise (*Pimpinella anisum*), and peppermint (*Mentha × piperita*). Castor (*Ricinus communis*) oil, ginger (*Zingiber officinale*), saffron (*Crocus sativus*), and senna (*Senna alexandrina*) mostly were not used by pregnant women. Moreover, cinnamon (*Cinnamomum verum*), anise (*P. anisum*), peppermint (*M. piperita*), and sage (*S. fruticosa*) were mostly used during lactation. Castor (*R. communis*) oil, ginger (*Z. officinale*), garlic (*Allium sativum*), and aloe (*Aloe vera*) mostly were not used during lactation. This study is of great importance in order to decrease the possibility of endangering the lives of fetuses and infants. A combined effort among researchers, scientists, lactating women, and pregnant women may help in changing wrong uses and thoughts about medicinal plants and help to improve the overall health of both mother and fetus.

## Introduction

Palestine is a holy land for Christians, Jews, and Muslims, and many civilizations have passed through this land over time (Canaanite, British, Jewish, Roman, etc.). This has led to the exposure todiverse cultures, especially in the use of medicinal plants, folkloric foods, and cosmetics. Due to the geographical location of Palestine in the Mediterranean region, it has many distinctive characteristics, including fertile soil and a moderate climate (Mendelssohn and Yom-Tov, 1999; Lev, 2006). In addition, Palestine was and still is a very important crossroad of trade between the East and the West, adding a special characteristic to the use of medicinal plants (Azaizeh et al., 2006; Azaizeh et al., 2010; Ben-Arye and Samuels, 2015), especially among pregnant and lactating women. Evidence of the ancient use of medicinal plants in the Mediterranean region was found in a cave from about 60,000 years ago (Saad et al., 2005).

In recent years, people in developing and advanced countries have used medicines that originate from natural sources to improve the quality of their life (Schoen et al., 2007). The use of plants during pregnancy and lactation differs from woman to woman due to the environment in which they live, their income, knowledge in phototherapeutic products, and side effects (Gluckman et al., 2015). Based on a literature review, there are few studies or clinical trials on the safety and efficacy of using herbs during pregnancy and lactation in the West Bank area of Palestine (Jaafari et al., 2016; Ahmed et al., 2017; Ung et al., 2017).

The use of plants by pregnant and lactating mothers without awareness of their harmful effects exposes both mother and fetus or baby to great dangers, like abortion, premature term, uterine bleeding, and physical and mental retardation of the fetus (Holst et al., 2009; John and Shantakumari, 2015). The most sensitive period in pregnancy is the embryogenesis stage, in which tissues differentiate and organogenesis occurs, so teratogenicity is higher (Dennery, 2007). Many physiological changes happen during pregnancy, which may cause nausea, vomiting, heartburn, constipation, hypertension, peripheral edema, hemorrhoids, and varicose veins (Moreira et al., 2014; Paupério et al., 2014).

In addition, there are changes during lactation, such as transient hair loss, postpartum depression, and changes in the skin (Beicovrtch, 1987). Because of these changes, pregnant and lactating women consider treating these issues with safe methods that avoid medications, which may include plants. The safest approach to provide substantial relief for common symptoms is using plants, which do not have strict regulations like modern medicine and are increasingly used (Lewis and Elvin-Lewis, 2003; Teschke and Eickhoff, 2015). Thus, it is important to understand the extent to which plant treatments are used, as well as the specific products used, the reason for which they are used, and factors that may predict which women are least likely to use them.

## Methods

An ethnopharmacological survey (cross-sectional observational design study) using a pre-piloted questionnaire (**Supplementary Material**) was undertaken on herbal remedies used by pregnant and lactating women in the West Bank area of Palestine (Tulkarm, Nablus, Tubas, Jenin, Qalqilya, Salfeit, Jericho, Ramallah, Bethlehem, Jerusalem, and Hebron). **Table 1** presents the socio-demographic characteristics of the participants, who were interviewed in the period from November 2017 to February 2018. A convenience sample method was used as an approach to the survey. The anonymous survey was piloted and reviewed in order to help in understanding, validating, clarifying, and producing a reliable study. The interviews were conducted by five trained researchers, who interviewed and collected the questionnaires from the study participants face to face in Arabic, the local language of the informants. The duration of the interviews ranged from 30 to 45 min, with one visit per interviewee in each case.

**Table 1:** Socio-demographic characteristics of the study population (N = 350).

Variables	N (%)
**Age (years)**	
Under 20	12 (3.4)
20–30	169 (48.3)
31–40	103 (29.4)
>40 years	66 (18.9)
**Educational level**	
Primary and illiterate	7 (2.0)
Middle school	55 (15.7)
High school	82 (23.4)
Graduate	164 (46.9)
Postgraduate	42 (12.0)
**Household income**	
High	91 (26.0)
Average	235 (67.1)
Low	24 (6.9)
**Place of residence**	
City	137 (39.1)
Village	209 (59.7)
Refugee camp	4 (1.2)
**Work**	
Yes	140 (40.0)
No (housewife)	210 (60.0)
**Number of children**	
First child	103 (29.5)
Second	78 (22.3)
Third	75 (21.4)
Fourth	32 (9.1)
Fifth	30 (8.6)
>5	32 (9.1)

*Data are presented as frequency (percent) from the total population studies (N = 350).

### Ethical Approval

The informed consent forms, ethics, and aims of the present study were reviewed and approved by the Institutional Review Board (IRB) at An-Najah National University (IRB archived number [20] October 2017).

The pre-piloted and validated questionnaires were administered through personal contact. This method is an effective and easy option for data collection. The objective of this survey was to obtain information on several issues, including (i) the names of plants commonly used during pregnancy and lactation, (ii) the period of pregnancy when the herbs are used, (iii) the methods of preparing the herbs, (iv) the reasons for using the herbs, and (v) problems that occurred during the use of these herbs. In most cases, the interviews often started in the form of informal discussions to achieve the confidence of the interviewees. The study was conducted in accordance with the current Good Clinical Practice (GCP) Guidelines (EME, 1997) and the requirements of the declarations of Helsinki (World Medical Association, 2008) and the International Conference on Harmonization (ICH, 1996) Guidelines. Written informed consent was obtained from the participants. In addition, the interviewed women signed a free, prior, and informed consent form, and to protect their interest, they were informed by the researchers in detail about the current study and its purposes. The women were not offered any incentives, and they were able to withdraw from the study at any time.

### Plant Identification

All of the plants were identified by the pharmacognosist Dr Nidal Jaradat after they were collected from the interviewees and kept in special glass frames. To verify and confirm the identity of each plant species stated by the interviewees, photographs and live specimens were used. Medicinal use was accepted as valid only if it was mentioned by at least three women. A herbarium specimen number was given for each sample of the collected herbs, as shown in **Table 2**, and voucher samples were kept at the Pharmacognosy Laboratory of the Department of Pharmacy, Faculty of Medicine and Health Sciences, An-Najah National University.

**Table 2:** Do you take herbs during your pregnancy and lactation.

Do you take herbs during your pregnancy and lactation?	N (%)
Yes	270 (77.1)
No	80 (22.9)
**Who recommended the herbs?**	
Myself	26 (7.4)
Family	88 (25.1)
Friends	176 (50.3)
Doctor	17 (4.9)
Pharmacist	21 (6.0)
Media and internet	22 (6.3)
**Reason preferring herbs**	
Safe than medication	236 (67.5)
Cost is less than medications	19 (5.4)
They are available	70 (20.0)
Others	25 (7.1)
**Benefits from herbs**	
Yes No	300 (85.7) 50 (14.4)
**Suffer from side effects**	
Yes	18 (5.1)
No	332 (95.9)
**Suffer from a miscarriage during pregnancy**	
Yes	42 (12.0)
No	308 (88.0)

*Data are presented as frequency (percent) from the total population studies (N = 350).

### Use Value

The use value (UV) is a quantitative value that can be used in order to show the relative importance of plant species known locally. It is calculated based on the following equation:

$$\text{UV}=\frac{\sum U}{n}$$

where UV is the use value of plant species, *n* is the number of informants, and *U* is the number of citations per plant species (Friedman et al., 1986).

### Family Use Values

The significance of medicinal plant families was assessed using the family use values (FUV), which were calculated according to the following equation:

$$FUV=\frac{UVs}{\left(ns\right)}$$

where UVs = use values of the taxa, and ns = total number of species within each family, which were used for the specific condition in the West Bank area of Palestine (Pieroni et al., 2004).

### Data Analysis

The Statistical Package for Social Sciences (SPSS version 17.0) and Microsoft Excel 2010 were used for the analysis of the data presented in this study. The significant association and the relation between different variables were evaluated using Pearson's correlation chi-square, with a significance level of p ≤ 0.05.

## Results

### Characteristics of Participants

Three hundred and fifty women were interviewed in the current survey, and questionnaires were completed. The socio-demographic characteristics are summarized in **Table 1**. The majority of respondents (48.3%) are 20–30 years of age and have various educational backgrounds, but most of them (46.9%) had a university education, while a minority (2.0%) of respondents was between the primary and illiterate levels of education. This ratio of illiterate versus university graduate women who participated in this study fits well with the most recent status of education in Palestine. Moreover, household income was sampled, and the majority of the participants have an average income (67.1%). In addition, most live in villages (59.7%), and 60.0% of the women who participated in the study are housewives. Knowledge about natural products was also investigated; indeed, the majority of the informants (77.1%) used herbs. Most of them (67.5%) used herbal plants because they believe they are safer than medications. **Table 2** shows that most mothers (67.5%) used the plants because they believe they are safer than medications during pregnancy and lactation, and most of them obtained information from relatives and friends (50.3%) and family members (25.1%).

In a survey regarding the use of herbal plants by pregnant and lactating women in Palestine, a total of 30 plant species distributed across 19 families were reported as locally traditionally used, which are presented in different tables according to their use. All families reported in this study are explained in **Tables 3**–**6**. Overall, the most commonly used FUV families were Apiaceae, Lamiaceae, and Leguminosae, respectively, with only one plant species reported for the remaining families.

**Table 3:** Medicinal plants used during pregnancy.

Plant names (Latin, English and Arabic names) with their voucher specimen codes	Family	Number of users (%)	Reason to use	Toxicity	Use value	FUV (%)
*Mentha × piperita* L./Peppermint/النعناع/Pharm-PCT-2812	Lamiaceae	217 (62.0)	Flatulence	Reported to cause jaundice in newborns and in some cases glucose deficiencies (Rita and Animesh, 2011)	0.62	30
*Salvia fruticosa* Mill/Sage/مريمية/Pharm-PCT-2117	Lamiaceae	205 (58.6)	Cramp	No clinical toxicological data support the toxicity effect, but studies linked its use with abortion	0.59	30
*Pimpinella anisum* L./Anise/اليانسون/Pharm-PCT-2768	Apiaceae	175 (50.0)	Sleep aid, Expectorant, Cold (flu)	Relatively safe (Sun et al., 2019)	0.50	20
*Camellia sinensis* (L.) Kuntze/Green teaاخضر/شاي/Pharm-PCT-2706	Theaceae	166 (47.4)	Health	Excessive intake induces liver toxicity (Bonkovsky, 2006)	0.47	10
*Anthemis cotula* L./Chamomile/البابونج/Pharm-PCT-178	Compositae	160 (45.7)	Sleep aid, Cold (flu)	Studies reported no evidence of acute toxicity (Pauli, 2008)	0.46	10
*Prunus dulcis* (Mill.) D.A.Webb/Almond/لوز/Pharm-PCT-143	Rosaceae	161 (46.0)	Heartburn	Safe and no observed adverse effect were observed (Song et al., 2010)	0.46	10
*Petroselinum crispum* (Mill.) Fuss/Parsley/بقدونس/Pharm-PCT-2739	Apiaceae	129 (36.9)	UT inflammation	No toxicity at low doses, but there should be caution in its administration to avoid overdosing (Wright et al., 2007)	0.37	20
*Coffea arabica* L./Coffee/قهوة/Pharm-PCT-2809	Rubiaceae	103 (29.4)	Stimulant	Reported to be nontoxic (Affonso et al., 2016)	0.29	10
*Allium sativum* L./Garlic/ثوم/Pharm-PCT-2704	Amaryllidaceae	67 (19.1)	Hypertension	No acute toxicity of garlic nor its constituents was reported (Chung, 2006)	0.19	10
*Cinnamomum verum* J.Presl/Cinnamon/قرفة/Pharm-PCT-2707	Lauraceae	61 (17.4)	Delivery aid	Toxicity data are insufficient. However, women used it to abort the pregnancy (Çolak et al., 2018)	0.17	10
*Rosmarinus officinalis* L./Rosemary/اكليل الجبل/Pharm-PCT-2732	Lamiaceae	60 (17.1)	Inflammation	Large amount of its oil is reported to cause nephritis and gastroenteritis (Al-Sereiti et al., 1999)	0.17	30
*Senna alexandrina* Mill/Senna/عشرج/Pharm-PCT-2808	Leguminosae	16 (4.6)	Laxative	Most of the published studies on senna genotoxicity studies reported negative results (Surh et al., 2013)	0.05	10
*Zingiber officinale* Roscoe/Ginger/زنجبيل/Pharm-PCT-2724	Zingiberaceae	28 (8.0)	Cold flu)	No studies reporting toxicity of ginger up to now (Mao et al., 2019)	0.08	10

**Table 4:** Medicinal plants not used during pregnancy.

Plant names (Latin, English and Arabic names) with their voucher specimen codes	Family	Number of users (%)	Reason for not using the plant	Toxicity	Use value	FUV (%)
*Ricinus communis* L./Castor/خروع/Pharm-PCT-2742	Euphorbiaceae	339 (96.9)	Abortion	Reported to have high toxic and pathogenic effects (Sarheed et al., 2018)	0.97	10
*Senna alexandrina* Mill/Senna/عشرج/Pharm-PCT-2808	Leguminosae	334 (95.4)	Diarrhea	Most of the published studies on senna genotoxicity studies reported negative results (Surh et al., 2013)	0.95	20
*Crocus sativus* L/Saffron/الزعفران/Pharm-PCT-2733	Iridaceae	325 (92.9)	Abortion	Saffron and its constituents are non-toxic in oral administration (Milajerdi et al., 2016)	0.93	10
*Zingiber officinale* Roscoe*/*Ginger*/*زنجبيل/Pharm-PCT-2724	Zingiberaceae	322 (92.0)	Burn Abortion	No studies reporting toxicity of ginger up to now (Mao et al., 2019)	0.92	10
*Aloe vera (L.) Burm.f*./Aloe/صبار/Pharm-PCT-115	Xanthorrhoeaceae	295 (84.3)	Constipation	Aloe contains multiple constituents with toxicological activities (Guo and Mei, 2016)	0.84	10
*Cinnamomum verum* J.Presl/Cinnamon/قرفة/Pharm-PCT-2707	Lauraceae	289 (82.6)	Abortion	Toxicity data are insufficient. However, women used it to abort the pregnancy (Çolak et al., 2018)	0.83	10
*Cuminum cyminum* L./Cumin/كمون/Pharm-PCT-2776	Apiaceae	279 (79.7)	Bleeding	Safety and lack of toxicity (Haddad et al., 2018)	0.80	20
*Trigonella arabica* Delile/Fenugreek/حلبه/Pharm-PCT-2511	Leguminosae	267 (76.3)	Early delivery	Studies showed a broad margin of safety for the standardized extract of fenugreek seeds (Kandhare et al., 2019)	0.76	20
*Coffea arabica* L./Coffee/قهوة/Pharm-PCT-2809	Rubiaceae	247 (70.6)	Nervousness Anemia	Reported to be nontoxic (Affonso et al., 2016)	0.71	10
*Petroselinum crispum* (Mill.) Fuss/Parsley/بقدونس/Pharm-PCT-2739	Apiaceae	221 (63.1)	Abortion	No toxicity at low doses, but there should be caution in its administration to avoid overdosing (Wright et al., 2007)	0.63	20
*Camellia sinensis (L.) Kuntze/*Green tea*/اخضر شاي /Pharm-PCT-2706*	Theaceae	184 (52.6)	Anemia	Excessive intake induces liver toxicity (Bonkovsky, 2006)	0.53	10
*Salvia fruticosa* Mill/Sage/مريمية/Pharm-PCT-2117	Lamiaceae	145 (41.4)	Abortion	No clinical toxicological data support the toxicity effect, but studies linked its use with abortion	0.41	10

**Table 5:** Medicinal plants used during lactation.

**Plant names (Latin, English and Arabic names) with their voucher specimen codes**	**Family**	**Number of users (%)**	**Reason for using the plant**	**Toxicity**	Use value	FUV (%)
*Trigonella arabica* Delile/Fenugreek/حلبه/Pharm-PCT-2511	Leguminosae	286 (81.7)	Inflammations Increase blood level, milk production	Studies showed a broad margin of safety for the standardized extract of fenugreek seeds (Kandhare et al., 2019)	0.82	11.11
*Cinnamomum verum* J.Presl/Cinnamon/قرفة/Pharm-PCT-2707	Lauraceae	157 (44.9)	Cramp Getting rid of blood after birth Increase milk	Toxicity data are insufficient. However, women used it to abort the pregnancy (Çolak et al., 2018)	0.45	11.11
*Salvia fruticosa* Mill/Sage/مريمية/Pharm-PCT-2117	Lamiaceae	138 (39.4)	Cramp sedation	No clinical toxicological data support the toxicity effect, but studies linked its use with abortion	0.39	22.22
*Pimpinella anisum* L./Anise/اليانسون/Pharm-PCT-2768	Apiaceae	121 (34.6)	Flatulence Cold (flu), sleeping aid	Relatively safe (Sun et al., 2019)	0.35	44.44
*Mentha × piperita* L./Peppermint/النعناع/Pharm-PCT-2812	Lamiaceae	134 (38.3)	Cramp Sedation, for good health	Reported to cause jaundice in newborns and in some cases glucose deficiencies (Rita and Animesh, 2011)	0.38	22.22
*Anthemis cotula* L./Chamomile/البابونج/Pharm-PCT-178	Compositae	96 (27.4)	For cough, sedation	Studies reported no evidence of acute toxicity (Pauli, 2008)	0.27	11.11
*Carum carvi* L./Caraway/كراوياء/Pharm-PCT-2779	Apiaceae	96 (27.4)	Increase blood level, milk production	Studies exhibited no acute toxicity and no significant side effect (Tabarraei et al., 2019)	0.27	44.44
*Prunus dulcis* (Mill.) D.A. Webb/Almond/لوز/Pharm-PCT-143	Rosaceae	91 (26.0)	For heartburn	Safe and no observed adverse effects were observed (Song et al., 2010)	0.26	11.11
*Camellia sinensis* (L.) Kuntze/Green tea/شاي اخضر/Pharm-PCT-2706	Theaceae	86 (24.6)	For good health	Excessive intake induces liver toxicity (Bonkovsky, 2006)	0.25	11.11
*Petroselinum crispum* (Mill.) Fuss/Parsley/نسبق دو/Pharm-PCT-2739	Apiaceae	75 (21.4)	Cramp, inflammation Increase blood level	No toxicity at low doses, but there should be caution in its administration to avoid overdosing (Wright et al., 2007)	0.21	44.44
*Coffea arabica* L./Coffee/قهوة/Pharm-PCT-2809	Rubiaceae	55 (15.7)	CNS stimulant	Reported to be nontoxic (Affonso et al., 2016)	0.16	11.11
*Cuminum cyminum* L./Cumin/كمون/Pharm-PCT-2776	Apiaceae	43 (12.3)	For flatulence	Safety and lack of toxicity (Haddad et al., 2018)	0.12	44.44
*Nigella arvensis* L./Nigella/قزحه/Pharm-PCT-1640	Ranunculaceae	48 (13.7)	Increase milk, enhance immunity	Its oil has low toxicity (Zaoui et al., 2002)	0.14	11.11

**Table 6:** Medicinal plants which cannot be used during lactation.

Plant names (Latin, English and Arabic names) with their voucher specimen codes	Family	Number of users (%)	Reason for not using the plant	Toxicity	Use value	FUV (%)
*Senna alexandrina Mill*/Senna/عشرج/Pharm-PCT-2808	Leguminosae	333 (4.9)	Diarrhea	Most of the published studies on senna genotoxicity studies reported negative results (Surh et al., 2013)	0.95	12.5
*Ricinus communis* L./Castor/خروع/Pharm-PCT-2742	Euphorbiaceae	328 (93.7)	Diarrhea	Reported to have high toxic and pathogenic effects (Sarheed et al., 2018)	0.94	12.5
*Aloe vera (L.) Burm.f./*Aloe/صبار/Pharm-PCT-115	Xanthorrhoeaceae	323 (92.3)	Constipation	Aloe contains multiple constituents with toxicological activities (Guo and Mei, 2016)	0.92	12.5
*Zingiber officinale* Roscoe*/*Ginger/زنجبيل/Pharm-PCT-2724	Zingiberaceae	317 (90.6)	Reduce milk production	No studies reporting toxicity of ginger up to now (Mao et al., 2019)	0.91	12.5
*Allium sativum* L./Garlic/ثوم/Pharm-PCT-2704	Amaryllidaceae	303 (86.6)	Bad taste	No acute toxicity of garlic nor its constituents was reported (Chung, 2006)	0.87	12.5
*Coffea arabica* L./Coffee/قهوة/Pharm-PCT-2809	Rubiaceae	295 (84.3)	Nervousness, reduce milk production	Reported to be nontoxic (Affonso et al., 2016)	0.84	12.5
*Petroselinum crispum* (Mill.) Fuss/Parsley/بقدونس/Pharm-PCT-2739	Apiaceae	275 (78.6)	Anemia	No toxicity at low doses, but there should be caution in its administration to avoid overdosing (Wright et al., 2007)	0.79	12.5
*Salvia fruticosa* Mill/Sage/مريمية/Pharm-PCT-2117	Lamiaceae	212 (60.4)	Reduce milk production	No clinical toxicological data support the toxicity effect, but studies linked its use with abortion	0.61	12.5

However, the current survey results revealed that peppermint (*Mentha × piperita*), sage (*Salvia fruticosa*), and anise (*Pimpinella anisum*) are the most used plants during pregnancy, with UV values of 0.62, 0.59, and 0.50, respectively, as presented in **Table 3**. **Table 4** shows that castor (*Ricinus communis*), senna (*Senna alexandrina*), and saffron (*Crocus sativus*) are the least used plants during pregnancy, with UV values of 0.97, 0.95, and 0.93, respectively. Moreover, **Table 5** shows that fenugreek (*Trigonella arabica*), cinnamon (*Cinnamomum verum*), and sage (*S. fruticosa*) are the most utilized plants during lactation, with UV values of 0.82, 0.45, and 0.39, respectively. Finally, **Table 6** reveals that senna (*S. alexandrina*), castor (*R. communis*), and aloe (*Aloe vera*) are the least utilized plants during lactation, with UV values of 0.95, 0.94, and 0.92, respectively.

### Plants Used or Not Used During Pregnancy

**Table 3** presents the plants that were cited by the pregnant participants with the number of users, the UV, and the reason for using the plant. Peppermint (*M. piperita*) was reported to be used to control flatulence and for calming, with the highest UV of 0.62, followed by sage (*S. fruticosa*) used for digestive problems, including cramps and loss of appetite, with a UV of 0.59. Other plants were reported for different uses, such as cinnamon (*C. verum*) reported to be used for delivery aid, and other herbs that may act as stimulants, such as coffee (*Coffea arabica*). Senna (*S. alexandrina*) was reported to be used as a laxative, with the lowest UV among other plants reported. In addition, ginger (*Zingiber officinale*), chamomile (*Anthemis cotula*), and anise (*P. anisum*) were reported to be used for the treatment of flu and its symptoms. Heath green tea (*Camellia sinensis*) was one of the best plants used. Garlic (*Allium sativum*) was reported to be used by participants for hypotension. Parsley (*Petroselinum crispum*) and rosemary (*Rosmarinus officinalis*) act as pain relievers with anti-inflammatory properties. Almond (*Prunus dulcis*) was reported to be used to prevent or reduce heartburn. Cinnamon (*C. verum*), senna (*S. alexandrina*), and ginger (*Z. officinale*) were infrequently reported by the participants and had the lowest UV, which was explained when they were presented with high UV in **Table 4**, which shows the least used plants by the participants.

Those with a negative attitude toward the use of medicinal plants suggested that herbal drugs are not pure or clean; they cause more adverse effects and have no clearly defined dosages, such that a few of the plants are included and presented in **Table 4**. Some plants should be avoided by pregnant women due to possible abortion-inducing effects, such as sage (*S. fruticosa*), cinnamon (*C. verum*), castor (*R. communis*), ginger (*Z. officinale*), parsley (*P. crispum*), and saffron (*C. sativus*). Castor (*R. communis*), senna (*S. alexandrina*), saffron (*C. sativus*), ginger (*Z. officinale*), and cinnamon (*C. verum*) were reported with the highest UV (0.97, 0.95, 0.93, 0.92, and 0.85, respectively), due to their direct influence on the pregnancy. Too much caffeine can lead to nervousness and iron deficiency, which can cause anemia. Moreover, caffeine isolated from coffee (*C. arabica*) and green tea (*C. sinensis*) can inhibit the absorption of calcium and iron. In addition, the use of too much senna (*S. alexandrina*) for the treatment of constipation may lead to diarrhea and spasm. Fenugreek (*Trigonella arabica*) is a plant that may cause early delivery. Other plants were reported for different reasons, while those reported as directly responsible for abortion were sage (*S. fruticosa*), cinnamon (*C. verum*), castor (*R. communis*), ginger (*Z. officinale*), fenugreek (*T. arabica*), parsley (*P. crispum*), and saffron (*C. sativus*).

### Plants Used or Not Used During Lactation

**Tables 5** and **6** present plants that were used or not used by lactating women. From **Table 5**, it is observed that most of the plants were used for similar reasons, such as increasing milk production, improving health by increasing the blood level, and helping to get rid of symptoms after birth. Cinnamon (*C. verum*), sage (*S. fruticosa*), peppermint (*M. piperita*), and anise (*P. anisum*) were the highest reported, with UV of 0.45, 0.39, 0.38, and 0.35, respectively. Cumin (*Cuminum cyminum*) had the lowest UV of 0.12. Sage (*S. fruticosa*), peppermint (*M. piperita*), cinnamon (*C. verum*), and parsley (*P. crispum*) were reported to be used for the treatment of cramps. For sleep induction, sage (*S. fruticosa*), peppermint (*M. piperita*), anise (*P. anisum*), and chamomile (*A. cotula*) were used. In addition, to increase milk production, women took caraway (*Carum carvi*), nigella (*Nigella arvensis*), cinnamon (*C. verum*), and fenugreek (*T. arabica*). To increase blood level, they used caraway (*C. carvi*), parsley (*P. crispum*), and fenugreek (*T. arabica*). For cold and flu, they took anise (*P. anisum*) and rosemary (*R. officinalis*). Chamomile (*A. cotula*) was also used as an antitussive, and nigella (*N. arvensis*) was used to boost immunity and to aid in getting rid of blood after birth.

**Table 6** shows the plants that cannot be used, mostly for the same reason, which directly or indirectly influence the production of milk. For example, they do not use coffee (*C. arabica*) because it causes nervousness and decreases milk. Castor (*R. communis*) and senna (*S. alexandrina*) cause diarrhea. Parsley (*P. crispum*) causes anemia. Fenugreek (*T. arabica*) makes the milk taste bad. Sage (*S. fruticosa*) and ginger (*Z. officinale*) were highlighted by the participants as reducing milk. Senna (*S. alexandrina*) and castor (*R. communis*) were reported with the highest UV of 0.95 and 0.94, respectively, for causing diarrhea, indirectly influencing the production of milk by the impact on overall health.

**Table 7** summarizes the parts used and the method of preparation of plants used during pregnancy and lactation. Seeds and leaves were the most used parts of all the plants. Boiling with water was the most common preparation method, while a few plants were eaten without boiling or other preparation methods.

**Table 7:** Plant products, parts used and preparation methods.

Plant	Plant part	Preparation method	%age of parts use
Sage	Leaves	Boil the leaves then take it orally.	30.8
Coffee	Seed	Boil the seed then take it orally.	38.5
Anise	Seed	Boil the seed then take it orally.	38.5
Castor	Oil	Take 20 ml of the oil orally.	7.7
Peppermint	Leaves	Boil the seed then take it orally.	30.8
Chamomile	Flower	Boil the dried flower then take it orally.	7.7
Caraway	Seed	Boil the dried seed then take it orally.	38.5
Nigella	Seed	Take the seeds orally or can be used as a food ingredient.	38.5
Almond	Seed	Take the seeds orally.	38.5
Cinnamon	Bark	Boil it then take it orally.	7.7
Tea	Leaves	Boil the dried leaves then take it orally.	30.8
Cumin	Seed	Boil 100 g ground seeds then take it orally.	38.5
Parsley	Leaves	Boil the fresh leaves then take it orally.	30.8
Fenugreek	Seed	Boil 100 g of the seeds then take it orally.	38.5

## Discussion

Usually, most of the communities used herbal remedies based on their experience or on the advice of relatives or friends. Therefore, it is necessary to find out the correct scientific evidence for the use, which may help form a correct understanding toward plants. From **Table 3**, it is concluded that anise (*P. anisum*), sage (*S. fruticosa*), and peppermint (*M. piperita*) were mostly used by pregnant women for the reasons that were mentioned earlier in the results and according to different studies, which found that peppermint (*M. piperita*) tea can be used to treat cramps (Rodriguez-Fragoso et al., 2008; Herro and Jacob, 2010; Taheri et al., 2011).

Anise (*P. anisum*) can be used for stomach upset (Liu et al., 2008) and intestinal gases (Myers et al., 2009) and as an expectorant (Gradinaru et al., 2014). According to **Table 4**, castor oil (*R. communis*), ginger (*Z. officinale*), saffron (*C. sativus*), and senna (*S. alexandrina*) mostly were not used during pregnancy. However, the most common reason for using castor oil (*R. communis*) during pregnancy is for labor induction (Kelly et al., 2013). Abortion occurs mostly during the first 24 h after taking castor oil (*R. communis*); common side effects are vomiting, diarrhea, and nausea. Nausea commonly occurs among pregnant women, and ginger (*Z. officinale*) can be used to solve this problem, but not for vomiting (Bryer, 2005).

Studies show that saffron (*C. sativus*) can adversely affect the growth of fetuses and induce several fetal malformations, noticeably skeletal malformations (Osgerby et al., 2002). Great caution should be taken when saffron (*C. sativus*) is used during pregnancy. Senna (*S. alexandrina*) has a strong effect, as it stimulates the uterus and affects fetal cell development. Long-term use, frequent use, or use of high doses has been linked to serious side effects, including laxative dependence and liver damage (Vanderperren et al., 2005).

As shown in **Table 5**, cinnamon (*C. verum*), anise (*P. anisum*), peppermint (*M. piperita*), and sage (*S. fruticosa*) were mostly used during lactation. Studies have affirmed that cinnamon (*C. verum*) has many benefits, including management of blood glucose levels (Hlebowicz et al., 2007), weight loss (Whitfield et al., 2016), detoxification (Xing et al., 2014), and increasing the amount of milk (Guler and Seker, 2009; Dog, 2009), but it should not be combined with fenugreek (*T. arabica*) because it can lower blood pressure (Talpur et al., 2005). Anise (*P. anisum*) can be used to increase the amount of milk (Kent et al., 2012), for constipation and irritable bowel syndrome (Mercadante et al., 2011; Mosaffa-Jahromi et al., 2016), and to induce sleep (Marinov and Valcheva-Kuzmanova, 2015). Peppermint (*M. piperita*) is mainly used to induce calming and relaxing effects (Cook and Lynch, 2008). Sage (*S. fruticosa*) is not used for increasing the amount of milk, but it can be used for abdominal pain, occasional nausea, and vomiting (Boone and Shields, 2005; Wilhelm et al., 2007).

There are contradictory opinions regarding the medicinal effect of parsley (*P. crispum*) and its effect as an abortifacient product. For example, in a study conducted on pigs, Chaudhary et al. (1986) found that photodermatitis occurred due to the presence of furocoumarins. Moreover, Awe and Banjoko (2013) reported that *P. crispum* ethanol leaf extract exhibited hepatotoxic and nephrotoxic activities. A study on *P. crispum* oil (0.6 ml/kg body weight) showed protective activity against zearalenone-induced reproductive toxicity and improved testosterone levels in mature male mice (Salah-Abbès et al., 2009). In addition, *P. crispum* ethanol seed extract (5 mg/kg) reduced the dysfunction in rat kidney caused by prostadin-induced abortion *via* immunohistochemical and immunofluorescent staining and biochemical analysis (Rezazad and Farokhi, 2014). Moreover, parsley (*P. crispum*) herb and root have approval status by the German Commission E for the urinary tract and kidney stones (Nathan and Scholten, 1999).

Apiol is the major component of parsley (*P. crispum*) essential oil, and its preparations have no legitimate use in therapeutics. The dangers associated with their use, especially in the haphazard and unrestricted dosages in attempts at abortion, have provoked legislation making such preparations available for sale by prescription only. It is common knowledge that this regulation is being flouted (Quinn et al., 1958). However, the apiol essential oil abortifacient effect has been documented in hospitals. For example, an unmarried primiparous 23-year-old woman was admitted to a hospital with an inevitable abortion. She menstruated regularly every 28 days, the last period being on June 15, 1973. She purchased a box of abortifacient capsules, which, according to the box, contained “Ferri carbonas saccharatus 2.55 gr; aloe 0.2 gr; tansy OS l gr; oleum pulegii 0.13 gr; apiol 0.13 gr; ferri oxydum praecipitatum rubrum 0.25 gr” (David and Randall, 1974). In fact, in medicine, apiole has been used for the treatment of menstrual disorders and as an abortifacient. It is an irritant, and, in high doses, it can cause liver and kidney damage. Cases of death due to attempted abortion using apiole have been reported (Amerio et al., 1968). The question of how apiole may cause abortion is unclarified; however, Rodolfo Marri, after conducting an experiment on isolated rabbit and guinea pig uteri, found an increase in strength and tone of contraction after the consumption of a small dose, while a large dose had a depressing effect on the uterus (Shorter, 2017).

Finally, **Table 6** shows most of the medicinal plants that are not used by lactating women. These plants were castor oil (*R. communis*), ginger (*Z. officinale*), garlic (*A. sativum*), and aloe (*A. vera*). Traditionally, lactating women do not use castor oil (*R. communis*), as it causes diarrhea, which they thought will influence milk production due to the loss of fluids and nutrition during diarrhea. This traditional knowledge is in line with the scientific fact that castor oil (*R. communis*) is usually used as a laxative (Umukoro and Ashorobi, 2005; Ezenwali et al., 2010), but scientifically, castor oil (*R. communis*) should not be used during lactation for a more serious reason, as castor seeds (*R. communis*) contain a toxic glycoprotein called ricin, which has the ability to agglutinate blood cells. It is taken into cells by endocytosis and causes acute cell death by inactivation of ribosomal RNA, inhibiting protein synthesis. Ricin is approximately 1,000-fold more toxic following parenteral administration or inhalation than by the oral route because of its destruction in the intestine (Alexander et al., 2008). The women do not use ginger (*Z. officinale*), as they think it reduces milk production, while a prior investigation shows that if ginger (*Z. officinale*) is used immediately postpartum, it can improve milk production (Paritakul et al., 2016). More studies are needed to determine the effect of ginger (*Z. officinale*) on milk production at greater than 3 days postpartum.

Human milk is not just a complex mixture of substances that best meets the nutritional requirements of the infant; the flavor of human milk is altered when lactating women eat sulfur-containing foods such as garlic (*A. sativum*). When human milk is flavored with garlic (*A. sativum*), infants breastfeed longer and suck more overall than they do when this flavor is absent, at least under conditions in which the mothers have been ingesting bland diets for several days. However, garlic (*A. sativum*) appears to be safe in amounts usually used in food preparation. Garlic (*A. sativum*) may change the smell of breast milk and affect a baby's feeding. There is no information on the safety of garlic (*A. sativum*) supplements in breastfeeding (Mennella and Beauchamp, 1993).

Aloe (*A. vera*) gel is used to help heal cracked nipples (Eghdampour et al., 2013; Saeidi et al., 2015); however, it is better to remove aloe (*A. vera*) gel from the nipple areas before feeding the baby, because the bitter taste of the aloe (*A. vera*) gel may influence the baby's feeding. Aloe (*A. vera*) latex is a yellow-colored liquid that comes from the inner skin of the aloe (*A. vera*) leaf. It can be taken orally in both dried and liquid forms. Aloe (*A. vera*) latex should be avoided, as it has a strong laxative effect (Sim et al., 2013).

## Conclusion

In conclusion, it is found that sometimes there were differences between traditional and scientific uses of herbal remedies during pregnancy and lactation. For example, traditional use of anise (*P. anisum*) differed greatly from its scientific uses for stomach upset and intestinal gases, as an expectorant, and to increase the amount of milk. In addition, ginger (*Z. officinale*) can be used for nausea, but a burning sensation prevents pregnant women from using it. Moreover, some herbal remedies should be avoided, like senna (*S. alexandrina*), which can stimulate the uterus and affect fetal cell development, and castor oil (*R. communis*), which leads to abortion. Traditionally, it is thought that saffron (*C. sativus*) can cause abortion, but it adversely affects the growth of the fetus and induces several malformations. Cinnamon (*C. verum*) is traditionally used for cramping, but scientifically it is used for weight loss, detoxification, and an increased amount of milk. Peppermint (*M. piperita*) is medicinally used for calming and relaxation, but there is no reason for using it traditionally. Sage (*S. fruticosa*) is used traditionally for cramping, and medicinally it can be taken for occasional nausea and vomiting. Ginger (*Z. officinale*) is traditionally prohibited, as it is thought to decrease milk production, but studies have showed that the immediate postpartum use of ginger (*Z. officinale*) can improve milk volume. Finally, garlic (*A. sativum*) was not used for its bad taste and smell, as it changes the smell of breast milk and affects the baby's feeding. Therefore, the community, especially pregnant and lactating women, should be educated regarding the correct usage of medicinal plants during pregnancy and lactation in order to decrease the possibility of endangering the lives of fetuses and infants.

## Data Availability Statement

All datasets generated for this study are included in the article/**Supplementary Material**.

## Ethics Statement

The studies involving human participants were reviewed and approved by the Institutional Review Board (IRB) - An-Najah National University. The patients/participants provided their written informed consent to participate in this study.

## Author Contributions

Study conception and design: AE. Acquisition, analysis and/or interpretation of data: AE, NJ. Final approval and overall responsibility for this published work: AE, NJ.

## Conflict of Interest

The authors declare that the research was conducted in the absence of any commercial or financial relationships that could be construed as a potential conflict of interest.
