# Supplementary material for: Public Knowledge, Attitude, and Practice on Herbal Remedies Used During Pregnancy and Lactation in West Bank Palestine
Source: Front Pharmacol. 2020 Feb 14;11:46. doi: 10.3389/fphar.2020.00046 (PMC7034419; doi:10.3389/fphar.2020.00046)
Supplement: Supplementary file 1 [file DataSheet_1.docx]

1. What is your age?

1) Under 20 2) 20 – 30 3) 31 – 40 4) >40

1. What is your educational level?

1) Primary and illiterate 2) Middle school 3) High school 4) Graduate 5) Postgraduate

1. Where do you live in Palestine?
2. City 2)Village 3) Camp
3. What is your family monthly income in?
4. High (2500 USD and above) 2) Average (1000 – 3000 USD) 3) Low (Below 1000 USD)
5. Do you work?
6. Yes 2) No (housewife)
7. Do you have any chronic diseases?
8. Yes 2) No if yes what?_........................_
9. How many children do you have?
10. This is my first child 2) second 3) Third 4) Fourth 5) Fifth 6) >5
11. Did you take any herb during your pregnancy/lactation?

1) Yes 2) No

1. Why did you prefer herbal products rather than traditional drugs?

1) They are safer than medications 2) Their cost is less than medications

3) They are available 4) Others ………. (Can choose more than one)

1. Who recommended you to take the herbal product?
2. No one (myself) 2) Family 3) Friends 4) Doctor 5)Pharmacist 6)media & internet
3. Did you perceive benefits from taking herbs?
4. Yes 2) No
5. Did you observe any possible side effects due to herbs use?

1) No 2) Yes If yes, like what?..........................................

1. Did you suffer from threatened miscarriage during this pregnancy?
2. Yes 2) No

# If your answer is yes, please answer the following questions:

| Method of Preparation | Problem occur | Reason | In which period  did you use herbs | Used or Not | Plants |  |
| --- | --- | --- | --- | --- | --- | --- |
|  |  |  |  |  | Sage | 1 |
|  |  |  |  |  | Cinnamon | 2 |
|  |  |  |  |  | Coffee | 3 |
|  |  |  |  |  | Senna | 4 |
|  |  |  |  |  | Castor | 5 |
|  |  |  |  |  | Ginger | 6 |
|  |  |  |  |  | Peppermint | 7 |
|  |  |  |  |  | Chamomile | 8 |
|  |  |  |  |  | Celery | 9 |
|  |  |  |  |  | Flax | 10 |
|  |  |  |  |  | Oats | 11 |
|  |  |  |  |  | Anise | 12 |
|  |  |  |  |  | Honey | 13 |
|  |  |  |  |  | Caraway | 14 |
|  |  |  |  |  | Tea | 15 |
|  |  |  |  |  | Cuminum | 16 |
|  |  |  |  |  | Garlic | 17 |
|  |  |  |  |  | Ginseng | 18 |
|  |  |  |  |  | حلبة | 19 |
|  |  |  |  |  | Ginkgo biloba | 20 |
|  |  |  |  |  | Echinacea | 21 |
|  |  |  |  |  | Raspberry | 22 |
|  |  |  |  |  | St. John wart | 23 |
|  |  |  |  |  | Fennel | 24 |
|  |  |  |  |  | Parsley | 25 |
|  |  |  |  |  | Licorice | 26 |
|  |  |  |  |  | Clove buds | 27 |
|  |  |  |  |  | Saffron | 28 |
|  |  |  |  |  | Nigella Setiva | 29 |
|  |  |  |  |  | Red Pepper | 30 |
|  |  |  |  |  | Almond | 31 |
|  |  |  |  |  | Nettles | 32 |
|  |  |  |  |  | Valerian | 33 |
|  |  |  |  |  | Rosemary | 34 |
|  |  |  |  |  | الصبر | 35 |
|  |  |  |  |  | Nutmeg | 36 |
|  |  |  |  |  | Basil | 37 |
|  |  |  |  |  | كتان | 38 |
|  |  |  |  |  | الجرجير الاخضر | 39 |
|  |  |  |  |  | Other plants | 40 |

# Plants not welling to use during pregnancy

| Why | Not used | Plants |  |
| --- | --- | --- | --- |
|  |  | Sage | 1 |
|  |  | Cinnamon | 2 |
|  |  | Coffee | 3 |
|  |  | Senna | 4 |
|  |  | Castor | 5 |
|  |  | Ginger | 6 |
|  |  | Peppermint | 7 |
|  |  | Chamomile | 8 |
|  |  | Celery | 9 |
|  |  | Flax | 10 |
|  |  | Oats | 11 |
|  |  | Anise | 12 |
|  |  | Honey | 13 |
|  |  | Caraway | 14 |
|  |  | Tea | 15 |
|  |  | Cuminum | 16 |
|  |  | Garlic | 17 |
|  |  | Ginseng | 18 |
|  |  | حلبة | 19 |
|  |  | Ginkgo biloba | 20 |
|  |  | Echinacea | 21 |
|  |  | Raspberry | 22 |
|  |  | St. John wart | 23 |
|  |  | Fennel | 24 |
|  |  | Parsley | 25 |
|  |  | Licorice | 26 |
|  |  | Clove buds | 27 |
|  |  | Saffron | 28 |
|  |  | Nigella Setiva | 29 |
|  |  | Red Pepper | 30 |
|  |  | Almond | 31 |
|  |  | Nettles | 32 |
|  |  | Valerian | 33 |
|  |  | Rosemary | 34 |
|  |  | الصبر | 35 |
|  |  | Nutmeg | 36 |
|  |  | Basil | 37 |
|  |  | كتان | 38 |
|  |  | الجرجير الاخضر | 39 |
|  |  | Other plants | 40 |

# Plant used during lactation

| Method of Preparation | Problem occur | Reason | Used or not | Plants |  |
| --- | --- | --- | --- | --- | --- |
|  |  |  |  | Sage | 1 |
|  |  |  |  | Cinnamon | 2 |
|  |  |  |  | Coffee | 3 |
|  |  |  |  | Senna | 4 |
|  |  |  |  | Castor | 5 |
|  |  |  |  | Ginger | 6 |
|  |  |  |  | Peppermint | 7 |
|  |  |  |  | Chamomile | 8 |
|  |  |  |  | Celery | 9 |
|  |  |  |  | Flax | 10 |
|  |  |  |  | Oats | 11 |
|  |  |  |  | Anise | 12 |
|  |  |  |  | Honey | 13 |
|  |  |  |  | Caraway | 14 |
|  |  |  |  | Tea | 15 |
|  |  |  |  | Cuminum | 16 |
|  |  |  |  | Garlic | 17 |
|  |  |  |  | Ginseng | 18 |
|  |  |  |  | حلبة | 19 |
|  |  |  |  | Ginkgo biloba | 20 |
|  |  |  |  | Echinacea | 21 |
|  |  |  |  | Raspberry | 22 |
|  |  |  |  | St. John wart | 23 |
|  |  |  |  | Fennel | 24 |
|  |  |  |  | Parsley | 25 |
|  |  |  |  | Licorice | 26 |
|  |  |  |  | Clove buds | 27 |
|  |  |  |  | Saffron | 28 |
|  |  |  |  | Nigella Setiva | 29 |
|  |  |  |  | Red Pepper | 30 |
|  |  |  |  | Almond | 31 |
|  |  |  |  | Nettles | 32 |
|  |  |  |  | Valerian | 33 |
|  |  |  |  | Rosemary | 34 |
|  |  |  |  | الصبر | 35 |
|  |  |  |  | Nutmeg | 36 |
|  |  |  |  | Basil | 37 |
|  |  |  |  | كتان | 38 |
|  |  |  |  | الجرجير الاخضر | 39 |
|  |  |  |  | Other plants | 40 |

# Plants not used during lactation

| Why | Not used | Plants |  |
| --- | --- | --- | --- |
|  |  | Sage | 1 |
|  |  | Cinnamon | 2 |
|  |  | Coffee | 3 |
|  |  | Senna | 4 |
|  |  | Castor | 5 |
|  |  | Ginger | 6 |
|  |  | Peppermint | 7 |
|  |  | Chamomile | 8 |
|  |  | Celery | 9 |
|  |  | Flax | 10 |
|  |  | Oats | 11 |
|  |  | Anise | 12 |
|  |  | Honey | 13 |
|  |  | Caraway | 14 |
|  |  | Tea | 15 |
|  |  | Cuminum | 16 |
|  |  | Garlic | 17 |
|  |  | Ginseng | 18 |
|  |  | حلبة | 19 |
|  |  | Ginkgo biloba | 20 |
|  |  | Echinacea | 21 |
|  |  | Raspberry | 22 |
|  |  | St. John wart | 23 |
|  |  | Fennel | 24 |
|  |  | Parsley | 25 |
|  |  | Licorice | 26 |
|  |  | Clove buds | 27 |
|  |  | Saffron | 28 |
|  |  | Nigella Setiva | 29 |
|  |  | Red Pepper | 30 |
|  |  | Almond | 31 |
|  |  | Nettles | 32 |
|  |  | Valerian | 33 |
|  |  | Rosemary | 34 |
|  |  | الصبر | 35 |
|  |  | Nutmeg | 36 |
|  |  | Basil | 37 |
|  |  | كتان | 38 |
|  |  | الجرجير الاخضر | 39 |
|  |  | Other plants | 40 |
